# Detection of severe injury on scene: mechanism of injury, telephone interrogation or ambulance crew assessment?

**DOI:** 10.1186/1757-7241-20-S1-O4

**Published:** 2012-03-22

**Authors:** I Wilmer, G Chalk, GE Davies, DJ Lockey

**Affiliations:** 1 London’s Air Ambulance, Barts and the London NHS Trust, London, UK

## Background

Accurate identification of serious injury is critical to the tasking of scarce resources like air ambulances and for the effective triage of injured patients to trauma centres. This service dispatches a doctor-paramedic team to major trauma patients. Dispatch is based on mechanism of injury (MOI); dispatching control room paramedic interrogation of caller (INT) or request after assessment by attending land ambulance crew (REQ).

## Aims

This study was conducted to identify which of the three dispatch methods was most effective at identifying patients with serious injury.

## Methods

A retrospective review of tasking data from January 2008 to December 2010 was undertaken. The type of dispatch was recorded for all patients. Appropriate dispatches were those that after attendance and assessment were considered serious enough to be escorted to hospital or where resuscitation was carried out on scene. Inaccurate dispatches were those that resulted in leaving the patient in the care of land ambulance crew after assessment.

## Results

2203 patients were attended in a 3 year period. Distribution of dispatches were MOI n= 417 (18.9 %), INT n= 1375(62.4%) and REQ n= 411(18.7%). Appropriate dispatch was MOI 58.7% (245/417), INT 69.7% (959/ 1375) and REQ 72.2% (297/411). INT and REQ were both significantly more accurate than MOI (p< 0.0001). There was no significant difference between INT and REQ (p= 0.36). Mean time to dispatch of the paramedic-physician team was: MOI= 4.1 min, INT= 8 min and REQ= 21 min.

## Conclusions

MOI is commonly used but the least accurate method of accurate tasking. Unstructured telephone interrogation by experienced paramedics is as successful as crew request after assessment on scene and both are significantly better than MOI. Telephone interrogation provides the improvement in accuracy of dispatch without the considerable delay seen with crew scene assessment.

